# Telemedicine strategy of the European Reference Network ITHACA for the diagnosis and management of patients with rare developmental disorders

**DOI:** 10.1186/s13023-020-1349-1

**Published:** 2020-04-25

**Authors:** Michael Smith, Elizabeth Alexander, Ruta Marcinkute, Dorica Dan, Myfanwy Rawson, Siddharth Banka, Jason Gavin, Hany Mina, Con Hennessy, Florence Riccardi, Francesca Clementina Radio, Marketa Havlovicova, Matteo Cassina, Adela Chirita Emandi, Melanie Fradin, Lianne Gompertz, Ann Nordgren, Rasa Traberg, Massimiliano Rossi, Aurelién Trimouille, Rasika Sowmyalakshmi, Bruno Dallapiccola, Alessandra Renieri, Laurence Faivre, Bronwyn Kerr, Alain Verloes, Jill Clayton-Smith, Sofia Douzgou, on behalf of ERN ITHACA

**Affiliations:** 1 Manchester Centre for Genomic Medicine, St Mary’s Hospital, Manchester University Hospitals NHS Foundation Trust, Manchester Academic Health Sciences Centre, Manchester, M13 9WL UK; 2 Department of Human and Medical Genetics, Institute of Biomedical Sciences of the Faculty of Medicine of Vilnius University, M. K. Čiurlionio g. 21/27, LT-03101 Vilnius, Lithuania; 3 Romanian National Alliance for Rare Diseases RONARD, 29 Avram Iancu, etaj III, 450143 Zalau, Romania; 4 Division of Evolution and Genomic Sciences, School of Biological Sciences, University of Manchester, Oxford Road, Manchester, M13 9PL UK; 5 European Commission, DG Health and Food Safety, Information Systems, Rue Breydel 4 / Breydelstraat 4, Building B232 – 1049, Brussels, Belgium; 6 Open Applications Consulting Ltd., Avoca House, 191 Parnell St, Rotunda, Dublin 1, Ireland; 7 Medical Genetics Department, La Timone Hospital, Marseilles Public University Hospital, 278 Rue Saint-Pierre, 13005 Marseille, France; 8 Genetics and Rare Diseases Research Division, Ospedale Pediatrico Bambino Gesù, IRCCS, 00146 Rome, Italy; 9 Department of Biology and Medical Genetics, Charles University 2nd Faculty of Medicine and University Hospital Motol, V Úvalu 84, 150 06 Prague 5, Czech Republic; 10 Clinical Genetics Unit, Department of Women’s and Children’s Health, University of Padova, Via Giustiniani, 3 - 35128 Padova, Italy; 11 Discipline of Genetics, Victor Babeș University of Medicine and Pharmacy, Piața Eftimie Murgu 2, 300041 Timișoara, Romania; 12 “Louis Turcanu” Clinical Emergency Hospital for Children, Strada Doctor Iosif Nemoianu 2, 300011 Timișoara, Romania; 13 Department of Medical Genetics, CHU de Rennes, 2 rue Henri Le Guilloux, 35033 Rennes cedex 9, France; 14 Department of Molecular Medicine and Surgery, Center for Molecular Medicine and Department of Clinical Genetics, Karolinska University Hospital, 171 77 Stockholm, Sweden; 15 Department of Genetics and Molecular Medicine, Hospital of Lithuanian University of Health Sciences Kauno klinikos, Eivenių Str. 2, LT-50161 Kaunas, Lithuania; 16 Department of Medical Genetics, CHU de Lyon, 162 Avenue Lacassagne, 69003 Lyon, France; 17 Genetic Department, Hospices Civils de Lyon and CRNL, GENDEV Team, INSERM U1028, U1028 / UMR 5292, Bd Pinel - 69677, Bron Cedex, France; 18 Department of Genetics, AP-HP Robert-Debré University Hospital, Bd Sérurier, 75019 Paris, France; 19 Medical Genetics, Department of Medical Biotechnologies, University of Siena, Policlinico Santa Maria alle Scotte, Viale Mario Bracci, 16, 53100 Siena, Italy; 20 Department of Medical Genetics and Centre of Reference for Developmental Anomalies and Malformative syndromes, CHU de Dijon, 14 Rue Paul Gaffarel, 21000 Dijon, France; 21 Université Paris Diderot, 5 Rue Thomas Mann, 75013 Paris, France

**Keywords:** European reference network, Developmental disorders, Telemedicine, Rare disease

## Abstract

**Background:**

The European Reference Networks, ERNs, are virtual networks for healthcare providers across Europe to collaborate and share expertise on complex or rare diseases and conditions. As part of the ERNs, the Clinical Patient Management System, CPMS, a secure digital platform, was developed to allow and facilitate web-based, clinical consultations between submitting clinicians and relevant international experts. The European Reference Network on Intellectual Disability, TeleHealth and Congenital Anomalies, ERN ITHACA, was formed to harness the clinical and diagnostic expertise in the sector of rare, multiple anomaly and/or intellectual disability syndromes, chromosome disorders and undiagnosed syndromic disorders. We present the first year results of CPMS use by ERN ITHACA as an example of a telemedicine strategy for the diagnosis and management of patients with rare developmental disorders.

**Results:**

ERN ITHACA ranked third in telemedicine activity amongst 24 European networks after 12 months of using the CPMS. Information about 28 very rare cases from 13 different centres across 7 countries was shared on the platform, with diagnostic or other management queries. Early interaction with patient support groups identified data protection as of primary importance in adopting digital platforms for patient diagnosis and care. The first launch of the CPMS was built to accommodate the needs of all ERNs. The ERN ITHACA telemedicine process highlighted a need to customise the CPMS with network-specific requirements. The results of this effort should enhance the CPMS utility for telemedicine services and ERN-specific care outcomes.

**Conclusions:**

We present the results of a long and fruitful process of interaction between the ERN ITHACA network lead team and EU officials, software developers and members of 38 EU clinical genetics centres to organise and coordinate direct e-healthcare through a secure, digital platform. The variability of the queries in just the first 28 cases submitted to the ERN ITHACA CPMS is a fair representation of the complexity and rarity of the patients referred, but also proof of the sophisticated and variable service that could be provided through a structured telemedicine approach for patients and families with rare developmental disorders. Web-based approaches are likely to result in increased accessibility to clinical genomic services.

## Background

Developments in telecommunications technology are enabling healthcare providers to consider the possibility of diagnosing and treating patients remotely [1]. Telemedicine has proven especially helpful in places where access to healthcare is difficult because of (a) the need to travel long distances to an expert centre and/or (b) the lack of specialised medical professionals locally [2, 3]. A common goal of telemedicine applications in various clinical specialties is to pave effective, efficient, and patient-friendly care pathways.

Telegenetics is the branch of telemedicine which uses an internet connection and web-based applications for clinical genetics services [4]. A systematic review by Hilgart et al. concluded that telegenetics is a useful adjunct to traditional genetics services [5]. Telegenetic services provide an opportunity for smaller or inexperienced centers to easily access the expertise more often present in large or central clinical genetics facilities [6, 7]. We previously described our experience of using a web-based process to facilitate access to a specialised clinical genetics service [8]. We showed that sharing patient medical records for expert review can lead to new diagnoses, the delineation of new gene-disease correlations, advice on clinical management and education of the participating experts [8–10].

The European Reference Networks (ERNs) were launched in March 2017 as virtual networks enabling healthcare providers across Europe to access and share expertise for the care of patients with complex or rare disorders [11]. The ERNs aim to facilitate the discussion and gather and synthesise expert opinion on rare conditions, presentations or complications. The scope is to attempt to shorten the diagnostic odyssey for patients and improve their clinical management. The European Reference Network on Intellectual Disability, TeleHealth and Congenital Anomalies (ERN ITHACA) was formed with the aim to harness available, international, diagnostic and clinical expertise in the sector of rare, multiple anomaly and/or intellectual disability syndromes, chromosome disorders and undiagnosed syndromic disorders for patient benefit.

The European Commission’s Directorate General for Health and Food Safety (DG SANTE) launched, on November 20th 2017, the First Clinical Patient Management System (CPMS). This is a web-based application aimed at supporting the ERNs in improving the diagnosis and treatment of rare or low prevalence complex diseases across national borders of Member States in Europe. The application was developed by software provider OpenApp [12].

The CPMS is a unique, European Union (EU)-wide, cross-border, digital platform where relevant professionals are able to share challenging cases with international experts with the aim of seeking advice or sharing knowledge for the benefit of both patients and colleagues. Prior to the CPMS use for clinical purposes, patient data protection criteria were defined by DG SANTE [13]. ERN leading teams are involved in the authorisation of specific members of the international experts’ community who are then provided with a CPMS-specific, password-protected login (2-way authentication). The consent process for patients was established by DG SANTE in collaboration with the legal and ethical working group of the ERNs. A common, information and consent form which complied with General Data Protection Regulation (GDPR) was agreed. The form was translated in 24 EU languages and is available to be used across all ERNs.

We present our experience of the telemedicine process of the ERN ITHACA for the first 12 months, focusing on initial trends, advantages and limitations of the system and challenges faced. Based on this experience we delineate our vision of the future use of telemedicine for patients with rare developmental disorders.

## Results

### Patient expectations

Table 1 outlines the themes and illustrative quotes identified by a focus group of ten patients and patient representatives as part of the 2017 EURORDIS - Rare Diseases Europe Alliance meeting held in Budapest.

**Table 1:** The outcomes of the EURORDIS ERN ITHACA focus group discussion

Theme	Description	Illustrative quotes
Rare is common	ERN ITHACA is distinctive in attempting to encompass diagnostic and care approaches for a large and variable number of rare conditions. The focus group felt that there is a lot of common ground to be found despite this variability.	*‘All rare diseases have something in common, we all have psychological issues, self-image, self-trust and psychosocial. You are part of the world, in your class, at work it has an impact, this is common, you’re limited, you’re not part of a group. How do we deal with this’*
		*‘The patient should have a unified ID, not the condition, because they are very different*
		*‘Added guidelines –‘what is the best thing to do for the child, what’s the best thing for the adult?’*
		*‘Clinical Guidelines don’t involve specific interventions for kids, I mean for individuals. Like physiotherapy, like music therapy. We talk a lot about diagnosis without treatment so I think we should include this type of guideline too’*
Access	There was a clear expectation that ERN ITHACA should improve patients’ access to experts for diagnosis but also provide guidance to other specialists for appropriate care and management.	‘*Patients need answers now, as soon as possible. I mean it takes time for change, if change takes place. It’s nice to plan them but we need answers as soon as possible, not in 5 years.’*
	Interestingly they also thought that the ERN should be an ‘information hub’ about rare diseases for the general public.	*‘I think it’s important for children with multiple birth defects that go from one clinician to another at a time, to have an authority such as the ERN to help solve the problem. This is the basic fact, it is not [just] that you have experts it that you have responsible use.’*
		*‘We are such a fantastic resource when it comes to training for all the medical professionals and to be included in their training is very much appreciated, I find’*
		*‘[the] main added value is the diagnostic aspect and probably to develop some sort of guidance to the centres how to communicate and how to ask for support in how to diagnose the cases.’*
		*‘Do the public know about the ERN, I mean not just the patients but the public.’*
Mapping	A key part of the discussion focused around the role of ERN ITHACA in mapping relevant services in different countries including specific deficiencies. The participants perceived this as a working plan for developing appropriate processes onwards.	*‘We should start with finding out which are the state of the art and to find out what kind of resources we have currently’*
		*‘Collect all the resources; guidelines, service use, training courses, leaflets, information, platforms, everything. A first step’*
		*‘We could map all existing sources of education and develop from what we have, because there are a lot of resources’.*
		*‘First you have to share the guidelines that are existing, after that you can see what’s missing. But I don’t think you can have one guideline for all the diseases, you have to have one guideline for each one and that’s a challenge how to go about that.’*
		*‘It’s a very important part, if it is developed into the ERN this type of guideline [supportive treatment] it should be more recognised at national level and services that usually are provided for therapy would be better recognised and more valued by healthcare systems.’*
Patient Centered approach	The group specifically expressed that patients and support groups should be major stakeholders in outlining the strategy of the ERN. This included rating the professionals involved; and teaching and training.	*‘This type of information [Annual Health checks] could tie into registry consent. Sometimes you feel abandoned, you would be able to update your information.’*
		‘*Expert centres [should be] appointed by patient organisations. Orphanet is nice but I think expert centres in the main hospitals should be on a list.’*
		*‘Experience shows everyone performs better if they are awarded. It’s good to evaluate, patient’s evaluate, the way they do for hotels, you would be surprised they may be knowledgeable but they mishandle people.’*
		‘*We should include training the other way to look at how you cope with chronic conditions’*
		*‘We should include training resources for the patient and family, to increase their resilience.’*
		*‘follow patients for many years and then share this with other patients to get an overview of this particular disease’*
		*‘…most rare diseases have psychosocial aspects too, it’s not mentioned anywhere, specialist physiotherapy.. it’s not just the same as physiotherapy. Education of the patients is very important in what to do.’*
		*‘I was thinking, I’m not sure if this fits but education of the parents with the new findings, that kind of thing.’*

Telemedicine processes in healthcare are usually adopted to allow for easier or faster access to relevant services. The inequality in securing access to professional help across different EU countries was emphasised by the focus group. This included access to expert clinical opinions, treatment options but also to standard information about rare developmental disorders; difficulties in accessing information about the psychological and social aspects of living with a rare disorder; the lack of information about access to ERN ITHACA. Communication was another recurrent theme and the focus group expressed their interest in sharing a platform with healthcare professionals to seek advice and share knowledge and experience. The concept of using telemedicine for the diagnosis and management of patients with rare developmental disorders was described as “very acceptable” with the caveat of ensuring patient data protection.

### ERN ITHACA clinical patient management system activity

#### Quantitative data

A total number of 28 cases from 13 different centres including one from a guest user were submitted for expert review using the ERN ITHACA CPMS by the end of the first year of operation (December 2018, Fig. 1). Analysis of the activity has been summarised in Fig. 2. Our analysis evidences that:

1. The online approach allowed doctors to easily reach an international expert group, regardless of the time of the day and geographical location. This is proven by observed participation of centres located in 50% of all participating countries (Fig. 2a). On the other hand, almost two thirds (66%) of ERN ITHACA centres (for a few countries there are multiple participating centres) did not contribute cases to the CPMS platform by the end of the first year. When asked for non-engagement reasons in an April–May 2018 survey commissioned by DG-SANTE (see Methods), some centres expressed difficulty in (a) getting national approval to use the CPMS because of patient data safety concerns on behalf of their national or local institutions and (b) acquiring the information technology skills to use the CPMS.
2. The submission rate was variable across the year (Fig. 2b) with reduced activity around regular holiday times. This is similar to non-web-based services [14] and so, in this case, a web-based system did not make significant differences to the distribution of referrals across a 12-month period. We noted that the submission rate and general activity of the participants was more dependent on reminders and newsletters than the time of the year itself.
3. There was variability in user type both for those submitting and those leading the panels (Fig. 2c). We noted a growing proportion of trainees and junior doctors submitting cases as time went by.

**Fig. 1:**
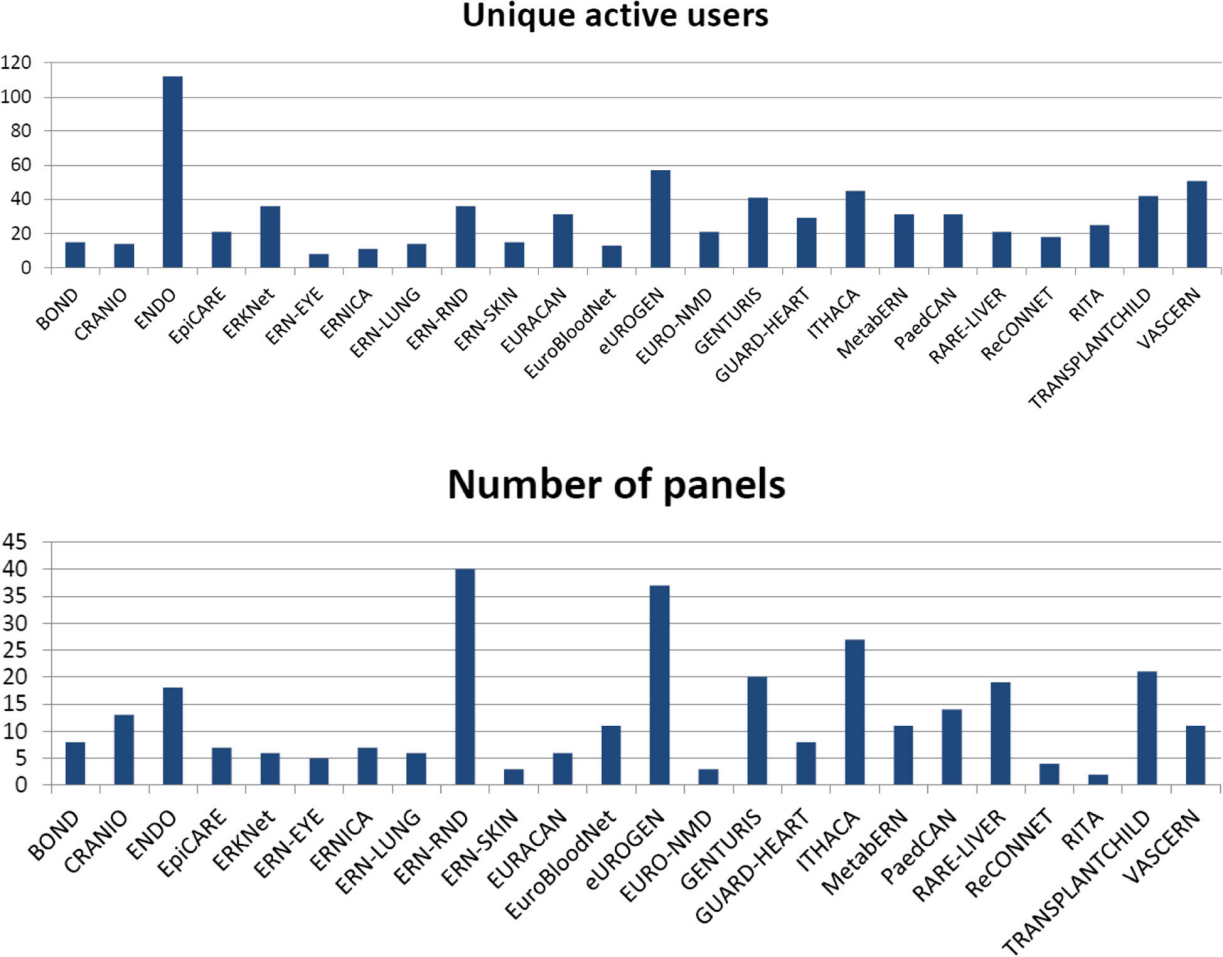
ERN CPMS activity measured in numbers of unique active users and numbers of submitted panels across different ERNs after 12 months of use

**Fig. 2:**
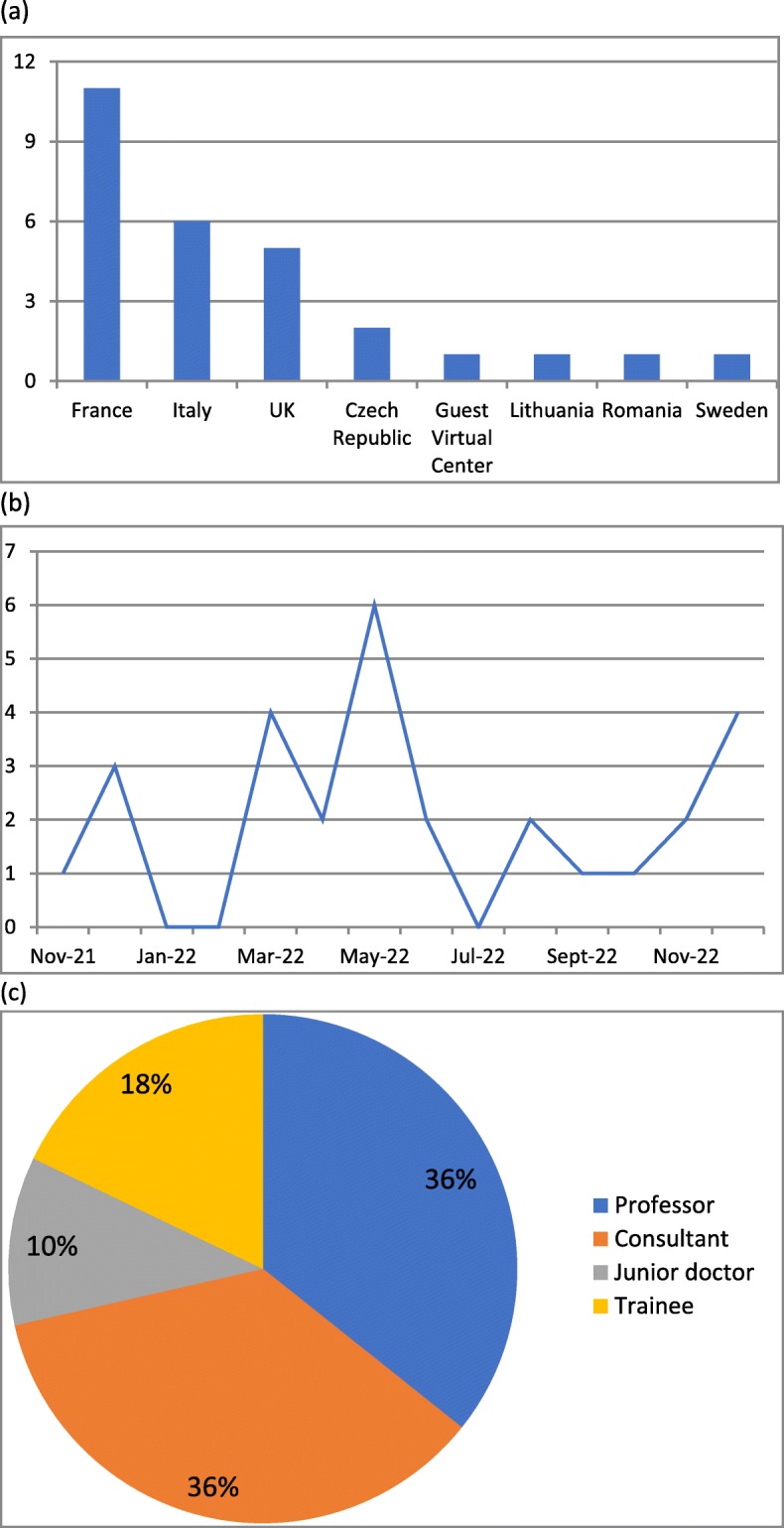
Indicators of the CPMS use, ERN ITHACA, 2018. **a** Countries that submitted cases to the CPMS, **b** The case submission rate across the year, **c** roles of people leading CPMS panels

#### Qualitative data

The queries about cases submitted to the CPMS were classified into groups relating to (1) diagnosis (phenotyping and/or genotyping) (2) requests of further testing as part of research and (3) clinical management. Of these, the greatest demand was diagnostic (27/28) with occasional queries about opportunities of recruitment to research (4/28) and clinical management (1/28). This is to be expected for an ERN which deals to a large extent with undiagnosed cases. Initially, referring clinicians tended to have a single query. However, the most recently submitted queries were multiple; doctors were seeking for advice regarding diagnosis of patients as well as options for participation in research.

**Fig. 3:**
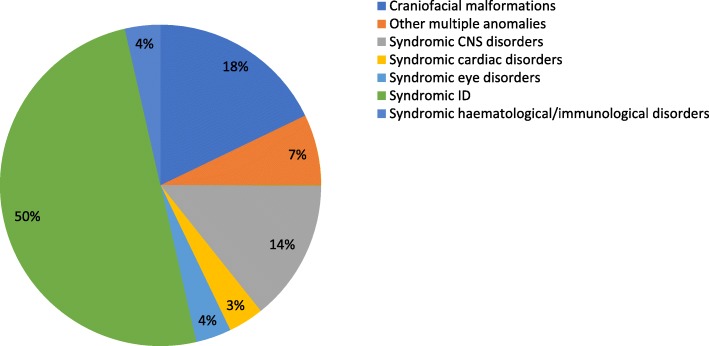
The distribution of submitted cases to the CPMS according to clinical area

The 28 submitted cases belonged to seven groups of developmental syndromes: (1) Craniofacial malformation (2) Other, multiple anomaly (3) Central nervous system (4) Cardiac (5) Eye (6) Intellectual disability (ID) and (7) Haematological/immunological. The distribution of submitted queries according to these areas is shown in Fig. 3. The vast majority of cases (14%) were related to syndromic ID, followed by craniofacial malformation syndromes (5%).

The number of participant comments on each panel query varied from 1 to 9 (mean: 4). Most of the replies were written by a single clinician, but there were several comments which in fact provided the consensus opinion of a group meeting of experts from a single centre. The diagnostic suggestions are specified in Table 2. In many cases these were requests for additional clinical information or suggesting further evaluation or genetic laboratory investigations.

**Table 2:** Diagnostic suggestions for the ERN ITHACA CPMS submitted cases (a) Syndromic groups and (b) distinct syndromes. For a few cases there were several suggestions

(a)			
Holoprosencephaly syndromes			
Chromatin remodelling disorders			
Blepharophymosis-ID syndromes			
RAS-MAPK pathway disorders			
Cutis laxa			
Mosaic disorders			
Neural crest defects			
Lamin spectrum disorders			
DNA breakage syndromes			
(b)			
**Syndrome name**	**Gene**	**Prevalence (Ref[**15**])**	**Mode of inheritance (Ref[**16**])**
Growth retardation with deafness and mental retardation	*IGF1*		AR
Fabry disease	*GLA*	1–5 / 10,000	XLR
Smith-Lemli-Opitz	*DHCR7*	Unknown	AR
Aarskog-Scott	*FGD1*	Unknown	XLR
De Barsy	*ALDH18A1*	< 1 / 1,000,000	AR
Myopathy, mitochondrial progressive, with congenital cataract, hearing loss, and developmental delay	*GFER*	Unknown	Mitochondrial
Syndromic microphthalmia	*SOX2*	< 1 / 1,000,000	AD
Autoimmune disease, multisystem, infantile-onset, 1	*STAT3*	< 1 / 1,000,000	AD
Borjeson-Forssman-Lehman	*PHF6*	< 1 / 1,000,000	XLR
Mental retardation, X-linked 93	*BRWD3*	Unknown	XLR
Hypomyelinating neuropathy, congenital, 3	*CNTNAP1*	Unknown	AR
CHARGE syndrome	*CHD7*	Unknown	AD
Cornelia de Lange syndrome 4	*RAD21*	1–9 / 100,000	AD
Epileptic encephalopathy, early infantile, 6 (Dravet syndrome)	*SCN1A*	Unknown	AD
Glycosylphosphatidylinositol biosynthesis defect 11	*PIGW*	Unknown	AR
Baller-Gerold s., Rothmund-Thomson s.	*RECQL4*	< 1 / 1,000,000	AR
LADD	*FGFR3, FGF10, FGFR2*	< 1 / 1,000,000	AD
ODDD	*GJA1*	Unknown	AD
Microphthalmia, isolated, with coloboma 8	*STRA6*	< 1 / 1,000,000	AR
Kunze	*TUBB*	Unknown	AD
Symmetric circumferential skin creases, congenital, 2	*MAPRE2*	Unknown	AD
Kabuki	*KMT2D*	1–9 / 100,000	AD
Di George	*22q11.2del (TBX1)*	1/4000	AD
Hemifacial microsomia		1–9 / 100,000	Sporadic, AD
PIK3CA-related overgrowth syndrome	*PIK3CA*	Unknown	Mosaic
Pfeiffer	*FGFR1, FGFR2*	< 1 / 1,000,000	AD
Restrictive dermopathy, lethal	*ZMPSTE24, LMNA*	< 1 / 1,000,000	AR
Hallermann-Streiff	Unknown	Unknown	Unknown
Cantu	*ABCC9*	< 1 / 1,000,000	AD
Pitt-Hopkins	*TCF4*	Unknown	AD
IQSEC2-related syndromic intellectual disability	*IQSEC2*	< 1 / 1,000,000	XL

### Handling variable queries

The CPMS queries regarding diagnosis of patients had two main themes: (1) patients without a known diagnosis and no plausible genetic test results or (2) patients with or without a diagnosis, but with a genetic variant of uncertain significance. The queries submitted included: What is the diagnosis? Is this the right diagnosis? Is the clinical diagnosis correct even if the laboratory result does not confirm it?

The queries in cases with a known diagnosis included: Have you seen a patient with a similar phenotype or a particular gene change before? Is this particular sign or symptom a part of this condition? Is this a good candidate for participation in a research project?

In a single instance there was a query for advice regarding management of a patient with a confirmed clinical and molecular diagnosis. The question asked was: ‘Should we use this type of medication in this patient?’ There was also a single genetic counseling query about the recurrence risk of the patient’s condition for other family members.

## Discussion

The telemedicine outcomes of ERN ITHACA represent the result of a long and fruitful process of interaction between the network lead team, EU officials, software developers and members of 38 EU genetics centers in an effort to organise and coordinate direct healthcare through a secure, web-based platform. After 12 months of use (December 2018), ERN ITHACA ranked third in telemedicine activity amongst 24 European networks: 28 very rare cases from 13 different health care providers in 7 countries were shared on the platform with a diagnostic, participation in research or clinical management query.

From the start, we delineated ERN ITHACA’s telemedicine goals with guidance and equal involvement from patient representatives. We believe that working together is crucial in providing high-quality and cost-effective care for the patients with rare or low-prevalence complex diseases we see in Genomic Medicine. Many of our patients have conditions involving multiple body systems and require social, psychological and medical input. Parent support group and patient involvement is crucial in outlining tailored, multidisciplinary, professional management throughout life. Here we highlight trends of patient support groups’ outline the common ground amongst patients with different rare developmental disorders; to improve access to relevant information and care; to develop new services; to invite and to include them as major stakeholders in all ERN-related activities. Future, prospective studies are necessary to assess the involvement and benefit of patient support groups in influencing the strategy of the ERNs.

Web-based approaches result in increased accessibility to genetic services. Although the small numbers of cases managed through the ERN ITHACA CPMS within the first 12 months do not allow for firm conclusions, our effort highlights that cases were reviewed remotely and expert opinions were offered independently from the location (or country) of the patient and the professional; expert, rare or very rare, clinical considerations were made for the first time; the suggestions made for diagnosis or differential diagnosis directed genetic testing and clinical follow-up; genetic counselling information was readily offered for the patient and family involved.

We believe that the variability of the queries in just the first 28 cases submitted to the ERN ITHACA CPMS are not only a fair representation of the complexity and rarity of the patients referred, but also proof of the sophisticated and variable service that could be provided through a telemedicine approach for patients with rare developmental disorders. The case-mix reflected the one usually seen in non-web-based genetics clinics. The spread of conditions highlights the breadth of clinical demand in the sector of rare developmental disorders. It also suggests that for many of the cases, co-operation with other rare disease ERNs would be beneficial for patients, as this would allow experts from several different disciplines to comment on the same cases. Also, a similar tendency to increasing complexity of queries over time has been observed previously in other telemedicine approaches [17].

The CPMS is a unique platform where doctors are able to share challenging cases with international experts with the aim of obtaining advice or sharing knowledge for the benefit of both patients and colleagues. The users included senior medical doctors but also trainees. We think that the time consuming process linked with CPMS submission may have limited senior clinician engagement. Furthermore, younger clinicians were more familiar with IT and less hesitant about engaging with telemedicine initiatives.

One of the main strengths of the CPMS is the particular attention paid to security of data, an aspect which is of paramount importance and is necessary for any future similar initiatives, as outlined by our patient support groups. However, local (specific to the local healthcare provider) or national approvals for the use of the CPMS were not always achieved and this was one of the most important hurdles in adopting and promoting CPMS use across the entire network. The responsibility of rolling out the CPMS, transversally, lies with the European Commission’s Directorate General for Health and Food Safety (DG SANTE) and was not a specific task of our team. We hope that our work highlights the utility of the CPMS. However the opportunity of using telemedicine to reach patients with rare developmental disorders can only be realistic if the CPMS can be safe and available for all stakeholders involved.

On the other hand, we also identified specific limitations in using the CPMS. Differently to other ERNs, ERN ITHACA mostly deals with diagnostic queries for unique, unsolved cases which is a time-consuming process. ERN ITHACA CPMS panels are usually open for a longer period of time as in-depth expert discussion is needed. We did not consider setting a time limit for case completion as productive because it does not always serve our diagnostic purpose or the long turnaround time of genomic testing. Moreover, the development of massive parallel sequencing techniques resulted in a specific healthcare need for the opinion of clinical geneticists regarding genomic variant interpretation [18]. Lastly, each case often requires a multidisciplinary approach. All the previous are standard tasks of the process of evaluation of a patient with a rare, developmental disorder. Overall, these tasks are highly clinically significant and cost-effective since each finding may improve diagnostics, prevention, support and tailor treatment and follow up for the investigated condition of intellectual disability. In the short-term, we can diagnose affected individuals in less time and provide genetic counseling, treatment and prevention options such as surveillance programs, psychological support, or preimplantation genetic diagnostics. In the long-term, more individuals with rare genetic syndromes will receive an accurate diagnosis and this will lead to more and better treatment and therapeutic options.

Our experience in using the CPMS highlighted that it was designed as an interface aimed to serve the general purposes of all ERNs. In the case or ERN ITHACA, the design of the CPMS interface did not capture the standard tasks that make part of the diagnostic process in a user-friendly way. We translated this into a restructuring request to the relevant DG SANTE team, in order to meet the specific needs of the ERN ITHACA user community. This is an on-going, customisation process that we hope will improve the experience of using the CPMS and impact positively on ERN ITHACA telemedicine outcomes in the long term. The results of this effort should enhance the CPMS utility for ERN-specific care outcomes in the long term.

Rare disease networks have always informally existed among relevant professionals in different countries within the EU and beyond. Notwithstanding this, the establishment of ERNs has been a step forward as it has signalled a formal attempt to synchronise professional efforts with patient need, safeguard a borderless transfer of medical information using electronic systems and create a future model for the sustainable care and management of patients with rare diseases.

## Conclusion

Our experience of using the ERN ITHACA CPMS for the first 12 months detailed initial outcomes, advantages and limitations of the system and challenges faced for patients with rare developmental disorders. We hope that the important lessons learned will benefit the ERN community and especially rare disease patients.

### Limitations

The small sample size in this study means that while any conclusions may be indicative of trends, they cannot be judged as definitive. This paper presents a preliminary analysis of the CPMS use by ERN ITHACA that should be read in the wider context of an overarching CPMS for all ERNs.

## Methods

### Eliciting patient expectations

To ascertain patient expectations, we organised a focus group of ten patients and patient representatives as part of a EURORDIS meeting held in Budapest in 2017. A topic guide was distributed to participants before the meeting and participants chose 5 topics for discussion which were formulated in the form of open questions:

- What do you think is the added value of ERN ITHACA?
- What are the views of the group about access to expertise?
- What are the groups’ views on education?
- How are we going to evaluate the ERNs activities?
- What are the views of the group on the benefits of patient registers?

The discussion was facilitated by the ERN ITHACA TeleHealth lead and a nominated lead from the patient representative group. The discussion was recorded following the consent of all participants. The recorded conversation was then transcribed. The transcription document was read and analysed by the TeleHealth lead of ERN ITHACA and a Genetic Counsellor experienced in qualitative analysis. The data was coded using a deductive approach and five emerging themes were identified.

### Submission process of a case to the ERN ITHACA CPMS

1. Registration process; 1.1) EU Login account creation 1.2) Request Access to CPMS and ERN 1.3) User validation by ERN coordinator: To submit a case to the ERN ITHACA CPMS, the user was a professional clinician either belonging to an ITHACA-affiliated healthcare provider or granted guest access by the network Coordinator. Before the case was submitted, the user was required to request access to the CPMS. There were two stages to this process: Firstly, the user registered for an EU login via a web-based form. Once this was approved by the central EU IT Help Desk, the user requested access to the ITHACA CPMS through the SAAS authentication system provided by the EU. After the request was authorised by the Coordinator or delegated deputy, the user logged-in to the ITHACA CPMS system via two-factor authentication (log-in device + code received via SMS to mobile phone or by mobile app).
2. On-boarding process into CPMS (User registration on CPMS to login via EU Login and two factor authentication mechanism: after the user accessed CPMS, a case was submitted by entering data fields directly on to the CPMS interface. The data requested by the system when uploading a case was both demographic and clinical in nature. The submitting user also had the opportunity to upload medical images/scans, draw a family pedigree using a web-based tool and enter free-text data summarising the clinical question or problem.
3. Patient enrolment process: Patient enrolment and model consent form for care, registries and contact for research activities; 4) Virtual consultation process: 4.1) Panel Request Form 4.2) Clinical, Genetic, Phenotypic and imaging Data entry 4.3) Panel Selection 4.4) Case assessment and collaboration 4.5) Panel outcome 4.6) Panel closure 4.7) Post-consultation survey: Once the data was finalised, the submitting user invited a ‘panel’ of other CPMS users to assess the case. Further information could be added to the case if requested by the panel members. The participants in the discussion panels were invited based on the specific interests which were self-declared upon application for ERN ITHACA membership.

### Sending out reminders

Daily digest email: users in active panels receive daily digest emails showing any outstanding tasks or actions to be taken during the virtual consultation process. Users were reminded to contribute feedback to submitted cases through automated email messages generated by the CPMS system itself. Users were automatically notified when they had outstanding tasks – in the case of a panel lead, this could be when the case was ready to progress to the next stage or for an invited panel contributor, when they had not recorded a contribution to a case or confirmed that they were satisfied with the data provided by the lead. All clinical ERN ITHACA network members were reminded to contribute to the CPMS via the monthly network newsletter.

### CPMS survey

On 5th April 2018 the branch of the European Commission which facilitates CPMS, DG-SANTE, sent a web survey to all European Reference Network Coordinators and those with CPMS approval rights. ERN ITHACA sent the survey to clinicians across the 37 health care providers which compose the network. The results of this survey were collated and distributed by DG-Sante on 4th June 2018.

## Data Availability

All data generated or analysed during this study is included in this published article.

## Abbreviations

CPMS: Clinical Patient Management System
DG SANTE: Directorate-General for Health and Food Safety
ERN ITHACA: European Reference Network on Intellectual Disability, TeleHealth and Congenital Anomalies
ERN: European Reference Network
EURORDIS: European Organisation for Rare Diseases
GDPR: General Data Protection Regulation
ID: Intellectual disability
